# Clinical and molecular epidemiology of veterinary blastomycosis in Wisconsin

**DOI:** 10.1186/1746-6148-9-84

**Published:** 2013-04-22

**Authors:** Jennifer L Anderson, Brian L Sloss, Jennifer K Meece

**Affiliations:** 1 Marshfield Clinic Research Foundation, Marshfield WI 54449, USA; 2 U.S. Geological Survey, Wisconsin Cooperative Fishery Research Unit, College of Natural Resources, University of Wisconsin-Stevens Point, Stevens Point WI 54481, USA

**Keywords:** Blastomycosis, Canine, Epidemiology, *Blastomyces dermatitidis*, Genetics

## Abstract

**Background:**

Several studies have shown that *Blastomyces dermatitidis*, the etiologic agent of blastomycosis, is a genetically diverse pathogen. Blastomycosis is a significant health issue in humans and other mammals. Veterinary and human isolates matched with epidemiological case data from the same geographic area and time period were used to determine: (i) if differences in genetic diversity and structure exist between clinical veterinary and human isolates of *B. dermatitidis* and (ii) if comparable epidemiologic features differ among veterinary and human blastomycosis cases.

**Results:**

Genetic typing of 301 clinical *B. dermatitidis* isolates produced 196 haplotypes (59 unique to veterinary isolates, 134 unique to human isolates, and 3 shared between canine and human isolates). Private allelic richness was higher in veterinary (median 2.27) compared to human isolates (median 1.14) (p = 0.005).

Concordant with previous studies, two distinct genetic groups were identified among all isolates. Genetic group assignment was different between human and veterinary isolates (*p* < 0.001), with more veterinary isolates assigned to Group 2.

The mean age of dogs diagnosed with blastomycosis was 6 years. Thirty cases were in male dogs (52%) and 24 were females (41%). The breed of dog was able to be retrieved in 38 of 58 cases with 19 (50%) being sporting breeds. Three of four felines infected with blastomycosis were domestic shorthair males between ages 6–12, and presented with disseminated disease. The other was a lynx with pulmonary disease. The equine isolate was from an 11-year-old male Halflinger with disseminated disease. Disseminated disease was reported more often in veterinary (62%) than human cases (19%) (*p* < 0.001).

**Conclusions:**

Isolates from all hosts clustered largely into previously identified genetic groups, with 3 haplotypes being shared between human and canine isolates confirming that *B. dermatitidis* isolates capable of infecting both species occur in nature. Allelic diversity measures trended higher in veterinary samples, with a higher number of total alleles and private alleles. Veterinary isolates of *B. dermatitidis* contributed a substantial amount of diversity to the overall population genetic structure demonstrating the importance of including veterinary isolates in genetic studies of evolution and virulence in this organism.

## Background

Blastomycosis is a significant health issue in humans and other mammals. This fungal infection often presents as a non-specific febrile illness that mimics viral and bacterial pneumonia, causing cough, fever, hospitalization and sometimes death. Moreover, dissemination of the infection to bone, skin, central nervous system and other organs is often observed. In dogs, it is thought to predominantly affect young, male, large sporting breeds, chiefly because of their propensity for outdoor activity [1-4]. Only Minnesota considers canine blastomycosis a reportable disease, therefore the number of cases occurring nationally is grossly underestimated. In Minnesota, canine cases occurred at 2.5 times that of humans from 1999–2009 [5]. Studies in Arkansas and Wisconsin report incidence of canine blastomycosis to be 10–13 times that of humans [6,7]. In highly endemic areas, incidence rates reach 1-2% annually, and in one 10 year retrospective, epidemiological study, a fatality rate of 41% was observed [3]. Although reported less frequently, blastomycosis infections in felines show similar symptomology and severity [8,9].

Several studies, have explored the genetic diversity of *Blastomyces dermatitidis*, the etiologic agent of blastomycosis [10-12]. More recently, genetic characterization using microsatellite markers has revealed that *B. dermatitidis* is a genetically diverse pathogen [13] with the 2 principally divergent genetic groups being associated with different clinical phenotypes in humans [14]. Previous studies have focused on the genetics of human clinical isolates as isolates from veterinary sources are less available for research. It is unknown whether canine, or other veterinary, clinical isolates of *B. dermatitidis* exhibit genetic diversity similar to human isolates or whether genetic groups could be associated with specific clinical phenotypes, as has been shown in humans.

Veterinary and human isolates matched with epidemiological case data from the same geographic area and time period were used to determine: (i) if differences in genetic diversity and structure exist between clinical veterinary and human isolates of *B. dermatitidis* and (ii) if comparable epidemiologic features differ among veterinary and human blastomycosis cases. The results of this study will provide a more complete understanding of the population genetic diversity of *B. dermatitidis* from a wider range of host species and assess the existence of host-specific and shared genotypes.

## Methods

### Isolates

The majority of isolates used in this study are part of the Marshfield Clinic Research Foundation’s *B. dermatitidis* bio-bank, which have been collected through our relationship with Marshfield Labs since 1999. Over this period Marshfield Clinic Research Foundation has obtained an extensive library of human and veterinary clinical isolates including 57 cultures of *B. dermatitidis* from canines, 4 from felines, and 1 equine isolate. In addition, we were gifted an isolate obtained from the stool of a dog diagnosed with acute blastomycosis [15]. The human and veterinary isolates used in this study were collected over the same time period and geographic area. It is unknown whether any isolates in this study were acquired from epidemiologically linked owners and pets, both diagnosed with blastomycosis.

### Genetic analysis

DNA was extracted from all veterinary *B. dermatitidis* isolates (n = 63) and genotyped with 27 microsatellite markers as previously described [13]. Up to 3 attempts were made for isolates that did not amplify initially for a given locus. Veterinary isolate genotyping data were combined with previously genotyped human isolates (n = 238). Rarefacted allelic and private allelic richness [16,17] were calculated with HPRARE [18] employing a rarefaction method to account for unequal sample sizes in the two isolate sets. Non-parametric Wilcoxon signed rank tests were used to compare genetic diversity estimates between human and veterinary isolates of *B. dermatitidis*.

Genetic analyses focused on delineating genetic structure, predicting clusters of related isolates, and comparing the diversity of strains across the different host species. Briefly, genetically identical isolates were identified using Genetic Analysis in Excel v6.41 [19] and subsumed to representative haplotypes. The program STRUCTURE [20] was used to predict the minimum number of genetic clusters (*K*) within the composite data, with analysis settings as previously described [14]. The method of Evanno *et al.*[21] as employed by Structure Harvester [22] was used to estimate the most likely *K* given the data in conjunction with the mean and variance of the ln probability of *K* across multiple replicates. Principle coordinate analysis (PCoA) of the standardized covariance of the haplotypic genetic distance as performed in Genetic Analysis in Excel v6.41 was used to analyse and graphically plot clusters of haplotypes. Isolates were assigned to genetic group based on haplotypic results from both STRUCTURE and PCoA analysis. A Chi-square test was used to assess difference in genetic group assignment between human and veterinary isolates.

### Medical record review

The test accession number was retrieved from each clinically confirmed isolate of *B. dermatitidis* and used to obtain the following information on each veterinary patient: city and state of veterinary clinic submitting the sample for testing; age, breed, and gender of the veterinary patient; and sample source. Location of infection was inferred from the sample source submitted for testing and cases were categorized as pulmonary only or disseminated infections (which were presumed to have originated as a respiratory infection). Samples submitted for culture that led to a classification of pulmonary disease included: tracheal or transtracheal wash, bronchial lavage, lung biopsy, and respiratory secretions. Samples classified as obtained from disseminated disease included biopsies or discharge collected from masses or lesions on any part of the body not considered part of the respiratory tract. Clinical information associated with each human isolate was obtained previously for another study [14]. A Chi-square test was used to assess differences in location of infection between human and veterinary cases.

## Results

### Genetic analysis

Typing of *B. dermatitidis* veterinary isolates (n = 63) across 27 loci yielded a 97.5% success rate. Forty-three alleles were unable to be determined as a result of either failed PCR or null alleles. Most of these undetermined alleles (n = 35) were from 3 isolates, obtained in 2001 and 2002. After genetic typing of veterinary isolates was completed and combined with previously typed human isolates, a total of 301 isolates represented 196 distinct haplotypes (59 unique to veterinary isolates, 134 unique to human isolates, and 3 shared between canine and human isolates). A summary of haplotypic allele diversity of *B. dermatitidis* isolated from veterinary and human hosts is shown in Table 1. The total number of alleles and private alleles identified in veterinary isolates was higher than those in human isolates (Table 1). The median (adjusted mean # of alleles/locus) rarefacted allelic richness of veterinary isolates and human isolates was 9.26 and 8.13, respectively. Testing the null hypothesis that overall allelic richness is the same in veterinary and human isolates showed no significant difference (Wilcoxon signed ranks 2-tailed, *p* = 0.1319). However, private allelic richness differed significantly between veterinary isolates (median = 2.27) and human isolates (median = 1.14) (Wilcoxon 2-tailed, *p* = 0.0050) with 20 of 27 loci having higher private allelic richness for veterinary versus human isolates. In addition, a subsequent 1-tailed Wilcoxon signed ranks test showed veterinary private allelic richness was greater than human private allelic richness (Wilcoxon 1-tailed, *p* = 0.0025).

**Table 1:** **Haplotypic allele diversity of *B. dermatitidis *isolated from veterinary and human hosts**

	**Veterinary haplotypes (n = 59)**		**Human haplotypes (n = 134)**		**Shared haplotypes (n = 3)**
**Locus**	**# of alleles**	**# of private alleles**	**# of alleles**	**# of private alleles**	**# of alleles**
**1**	4	1	3	0	2
**2**	8	0	8	0	1
**3**	13	3	12	2	2
**4**	10	3	7	0	2
**5**	8	4	5	1	1
**6**	14	3	13	2	3
**7**	9	3	7	1	2
**8**	9	2	10	3	1
**9**	7	2	6	1	2
**10**	9	1	9	1	1
**11**	12	3	11	2	1
**12**	8	0	10	2	2
**13**	8	0	8	0	1
**14**	9	0	9	0	1
**15**	7	0	8	1	1
**16**	12	3	9	0	1
**17**	5	1	6	2	1
**18**	11	1	14	4	1
**19**	9	3	11	5	2
**20**	12	5	10	3	1
**21**	12	3	10	1	1
**22**	11	3	10	2	1
**23**	7	2	6	1	1
**24**	7	3	5	1	1
**25**	6	0	8	2	1
**26**	13	5	9	1	2
**27**	10	1	10	1	2
**Totals**	**250**	**55**	**234**	**39**	**39**

Summary of haplotypic allele diversity of *B. dermatitidis* isolated from veterinary and human hosts. The 301 clinical *B. dermatitidis* isolates were subsumed to 196 unique haplotypes; 59 unique to veterinary isolates, 134 unique to human isolates, and 3 shared between veterinary and human isolates.

**Figure 1:**
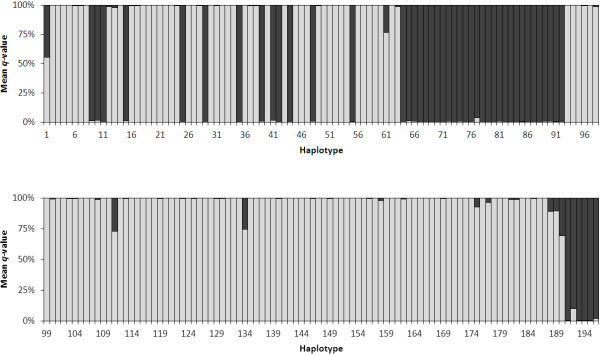
**STRUCTURE analysis of *B. dermatitidis *haplotypes.** Mean *q*-values from STRUCTURE for all 196 unique haplotypes (representing 301 isolates) with K = 2. Group 1 haplotypes are shown in black and Group 2 haplotypes in gray. Haplotypes 1–54 represent exclusively canine isolates. Haplotype 55 represents the single equine isolate. Haplotypes 56–59 represent feline isolates. Haplotypes 61–87, 89–91, 93–194, and 196 represent exclusively human isolates. Haplotypes 88, 92, and 195 contained both human and canine isolates. Five haplotypes (1, 61, 111, 134, and 190, representing only one isolate each) had a majority q-value <80% and could not be assigned to either genetic group.

**Figure 2:**
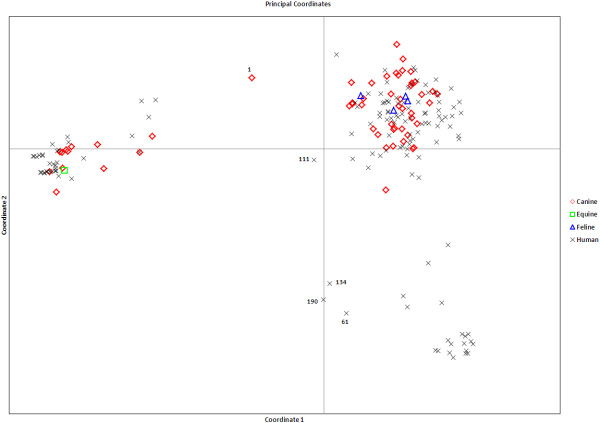
**Principle coordinate analysis of *B. dermatitidis *haplotypes.** Principle coordinate analysis of the haplotypic pairwise covariance distance matrix for all 196 unique haplotypes (representing 301 isolates). Fifty percent of variance was accounted for by the first principle coordinate (coordinate 1) and separated Group 1 and 2 isolates. The five numbered haplotypes represent single isolates with a mean majority *q*-values <80% in STRUCTURE results and could not be assigned to either genetic group.

STRUCTURE analysis of the unique haplotypes resolved two genetic clusters (*K* = 2) in the data based on the method of Evanno et al. [21] and the linearity and variance of lnP(D). The individual ancestry of each haplotype (q-value) based on *K* = 2 revealed 191 haplotypes with a majority q-value >80% (Figure 1). Five haplotypes (1, 61, 111, 134, and 190, representing only one isolate each) had a majority q-value <80%. Principal coordinate analysis (Figure 2) confirmed clustering of the haplotypes, primarily, into 2 distinct groups. Fifty percent of variance was accounted for by the first principle coordinate (Coordinate 1) and separated Group 1 and 2 isolates. The secondary axis (Coordinate 2) explained an additional 16% of variance. The 5 haplotypes (numbered in Figure 2) with <80% ancestral membership in STRUCTURE analysis, were observed to be genetic intermediates by PCoA analysis and could not be assigned to either genetic group. After removing genetic intermediates (1 canine isolate and 4 human isolates), Group 1 contained 48 haplotypes (representing 17 veterinary isolates and 135 human isolates). Group 2 contained 143 haplotypes (representing 45 veterinary isolates and 99 human isolates). Testing the null hypothesis that group assignment is the same for veterinary and human isolates showed that significantly more canine isolates were assigned to Group 2 (chi-square = 17.98, 1 df, *p* < 0.001). Although not statistically supported by STRUCTURE analysis as a separate genetic group, visual examination of Figure 2 reveals a small cluster of exclusively human isolates within Group 2.

### Medical record review

Samples were submitted for either fungal or aerobic culture testing by 42 veterinary clinics in 34 cities throughout Wisconsin, Illinois, Michigan and Minnesota. The highest numbers of samples submitted for culture over the 13 year period were received from veterinary clinics in Buffalo Grove, IL (n = 6), Plover, WI (n = 5), and Appleton, WI (n = 5). The age range of dogs diagnosed with blastomycosis was 1–17 years (mean 6), with 8 cases having unspecified ages. Thirty cases were male dogs (52%), 24 were female (41%), and 4 had unspecified gender (7%). The highest numbers of isolates were obtained in 2006, 2007, and 2009 with 9, 13, and 8 isolates cultured, respectively, in each of those years. The breed of dog was able to be retrieved in 38 of 58 cases with 19 (50%) being purebred sporting breeds (Table 2). Three of the 4 felines were domestic shorthair (*Felis catus*) neutered males between the ages of 6–12 and presented with disseminated disease. The other was a lynx (*Lynx canadensis*), which presented with pulmonary disease, and its age and gender were not specified. The equine isolate was obtained from an 11-year-old Halflinger neutered male (*Equus ferus caballus*) and was the result of disseminated disease.

**Table 2:** Breeds of 58 canines diagnosed with blastomycosis

**Breed**	**No. of dogs**
Labrador retriever^1^	9
Golden retriever^1^	5
Mixed^2^	4
German shepard	4
Cocker spaniel^1^	3
Shih tzu	2
Other^3^	11
Unspecified	20
**Total**	**58**

^1^breeds belonging to AKC sporting group.

^2^Mixed breeds included 1 mixed Doberman, 1 mixed Labrador retriever,

1 mixed Golden retriever, and 1 mixed Rottweiler/German shepard.

^3^Other includes one each of the following breeds: Alaskan malamute, Beagle, Australian cattle dog, Chow chow, German shorthaired^1^, Siberian husky, Jack russell terrier, Shetland sheepdog, Treeing walker coonhound, Weimaraner^1^, and Yorkshire terrier.

Twenty-one (33%) veterinary infections were identified as pulmonary, whereas 39 (62%) were classified as disseminated infections. Three infections were unable to be classified based on available information. The location of infection in human cases, abstracted from medical charts as previous described, showed 190 (80%) were pulmonary, 46 (19%) were disseminated, and 2 (< 1%) infections had undetermined location [14]. Testing the null hypothesis that location of infection is the same in veterinary and human cases showed a significant difference in the number of pulmonary and disseminated infections between human and veterinary cases (chi-square = 48.399, 1 df, *p* < 0.001).

## Discussion

Studies exploring the genetic diversity of *B. dermatitidis* have primarily focused on human clinical isolates as clinical isolates obtained from veterinary sources are less available to researchers. It is unknown whether canine, or other veterinary, clinical isolates of *B. dermatitidis* exhibit genetic diversity similar to human isolates or whether genetic groups could be associated with specific clinical phenotypes, as has been shown in humans [14]. Understanding strain level phenotypic variation across host species could help us better anticipate clinical symptoms and outcomes and potentially inform treatment options.

Genotyping of veterinary isolates with these previously published microsatellite markers continued to provide a reliable and informative tool for assessing population level diversity. Compared to human isolates, the veterinary isolates had a slightly lower genotyping success rate. Most of the missing genotype data came from 3 of the oldest isolates in our collection (obtained in 2001 and 2002) and are likely the result of DNA degradation. The remaining missing data points are across isolates and markers and likely represent genuine alleles (null alleles) especially since repeated attempts were made to amplify missing alleles. Furthermore these markers were initially designed from genomic sequence of a human *B. dermatitidis* isolate [13]. Given the larger number of total and private alleles detected in canine isolates of *B. dermatitidis*, it is probable that alleles exist in canine isolates that the primers sets used in this study cannot detect. Missing alleles are not reported in counts of the number of alleles or the number of private alleles shown in Table 1. However isolates with missing alleles were left in the haplotypic analysis.

In the current study, PCoA and STRUCTURE analyses resolved veterinary and human isolates intermixed within previously identified genetic groups with no distinct resolution of veterinary isolates discrete from human isolates. Allelic diversity measures trended higher in veterinary samples, with a higher number of total and private alleles, despite the more than two-fold higher number of haplotypes in the human isolates. Three haplotypes were shared between human and canine isolates confirming that *B. dermatitidis* isolates capable of infecting both humans and canines occur in nature. Despite this, a large number of alleles (94 of 289, 33%) and most haplotypes (193 of 196, 98%) were not shared between host species. In fact, all four feline isolates and the single equine isolate comprise unique haplotypes individually. One notable observational finding is the presence of a small genetic cluster within Group 2 that is exclusively comprised of human isolates. Veterinary isolates of *B. dermatitidis* contributed a substantial amount of diversity to the overall population genetic structure demonstrating the importance of including veterinary isolates in genetic studies of evolution and virulence in this organism.

*B. dermatitidis* isolates exhibit considerable population genetic diversity by microsatellite typing. This is especially true of Group 2 isolates regardless of the host species from which it is isolated. This may indicate that other factors, such as genetic recombination rates and ecological or environmental partitioning, may impact the population genetic structure of *B. dermatitidis* as has been observed in the closely related fungal pathogens *Coccidioides* and *Penicillium marneffei*[23,24]. Very little work has been done exploring genetic recombination in *B. dermatitidis*. Although *B. dermatitidis* can reproduce sexually, the mating-type locus was only recently identified [25], and it is unknown how frequently sexual reproduction occurs in nature. The rate of recombination between and among the 2 major genetic groups of *B. dermatitidis* could significantly impact the population genetic structure of this pathogen. Further exploration of ecological partitioning could also provide interesting insight into the genetic structure and evolution of *B. dermatitidis*. Mapping of exposures and the identification of point-source outbreaks have provided clues about the growth requirements and preferred environmental conditions of *B. dermatitidis*[26,27]. Although no apparent geospatial relationships have been identified in the distribution of *B. dermatitidis*, this work has been previously hindered by the limited number of environmental isolates and cultured clinical isolates with associated exposure data. It is possible that spatial partitioning exists on a more subtle level and is impacting the genetic diversity of *B. dermatitidis*. It is also recognized that *B. dermatitidis* exists in the environment in geographically-restricted microfoci that are apparently short-lived [28]. This may complicate attempts at fully understanding spatial relationships as time and seasonality may also play a role [29].

Another explanation of the varying levels of genetic diversity between host species observed in this study is that they are the result of host sampling. Canines and felines are exposed to a wider range of environments and in more direct environmental contact than humans. For example, the amount of direct contact and exposure of dogs (both surface area covered and time exposed directly to that surface) to the moist soil environments thought to be the primary habitat of *B. dermatitidis* is immeasurably higher than that of even the most-avid outdoorsman. Thus, dogs should be a better representative or sampling vector of the species diversity than the limited exposures available in human isolates. This is especially relevant to researchers as environmental isolates are rare. As seen in previous epidemiologic studies of canine blastomycosis, we observed a high rate of infection in male, sporting breed dogs. Unexpectedly, we found that the mean age of infected dogs in our study was higher than in previous reports [2-4,7].

Veterinary isolates were obtained more often from cases of disseminated disease versus pulmonary disease. This may be due to delay of diagnosis in dogs as owners may fail to notice the initial symptoms of blastomycosis, which can present as a non-specific febrile illness. Alternatively, it may be the result of intrinsic disease differences in varying host species, unrelated to strain type. One difference in disease manifestation that has been observed between humans and dogs is the higher rate of eye dissemination in dogs, which to date, has remained unexplained. However, the higher rate of dissemination in dogs may be a bias of our study associated with the ease of obtaining a biopsy in a disseminated case versus a sample such as a bronchial wash, which would be necessary in the case of pulmonary only disease presentation. Due to this, we felt confident classifying the disease location in veterinary cases based on the assumption that a veterinarian would not submit a pulmonary sample if another sample could be obtained with a less invasive procedure. Therefore, any isolates for which we had a non-respiratory sample were considered disseminated infections. Samples that were unable to be classified as either disseminated or pulmonary infections included one unidentified sample and two obtained from the nares. As blastomycosis is acquired by inhalation of spores, it is possible that an infection in the nares could be due to direct inoculation instead of dissemination from the lung and therefore we chose not to classify these cases as disseminated infections.

Another interesting finding of this study was that significantly more veterinary isolates (45 of 62, 73%), than human isolates (99 of 234, 42%) were genetic Group 2 organisms. In a recently conducted study, we found that genetic Group 2 infections are more often associated with disseminated disease in humans [14]. We were unable to directly assess association between genetic group and location of infection in veterinary cases in this study, because of the small number of Group 1 isolates. Despite this challenge, we observed a greater number of cases of disseminated disease in our veterinary cohort, with the predominant genetic group being Group 2 in these isolates. This is consistent with the associations established in the aforementioned study. If these results can be definitively verified across host species, it may indicate a molecular basis for differences in clinical phenotype observed between the two genetic groups.

Despite the patterns and correlations observed in this study, several limitations should be addressed in future research. The statistical power of this study is limited by the relatively small number of veterinary isolates and retrospective nature. Although culture is the primary diagnostic method for human blastomycosis, this may not be true of canine blastomycosis. This is supported by the fact that only 57 cultures of *B. dermatitidis* were isolated from canine sources by Marshfield Labs from 1999–2011. Certainly this collection of cultured isolates does not represent the clinical or genetic spectrum of canine blastomycosis, as veterinarians may be more likely to order culture under specific clinical circumstances such as more severe disease, obvious dissemination, value of pet, and a myriad of other factors. By partnering with veterinarians to obtain a less biased collection of isolates, future research can further explore the genetic diversity of veterinary blastomycosis. Although all clinical veterinary *B. dermatitidis* cultures used in this study were isolated and diagnosed in the same laboratory, over the same period of time and were obtained from the same geographic range as the human isolates to which they were compared, it must be recognized that this collection of isolates represents a limited geographic range of this global species. Despite the small number of veterinary isolates evaluated, this manuscript represents the largest genotyping study of canine *B. dermatitidis* isolates.

## Conclusions

Genetic typing and comparison of veterinary and human isolates revealed that they were intermixed within previously identified genetic groups with no distinct resolution of veterinary isolates discrete from human isolates. Allelic diversity measures trended higher in veterinary samples, with a higher number of total and private alleles. Three haplotypes were identified as shared between human and canine isolates confirming that *B. dermatitidis* isolates capable of infecting both humans and canines occur in nature. Veterinary isolates of *B. dermatitidis* contributed a substantial amount of diversity to the overall population genetic structure demonstrating the importance of including veterinary isolates in genetic studies of evolution and virulence in this organism.

Another finding of this study was that significantly more veterinary isolates (73%), than human isolates (42%) were genetic Group 2 organisms and cultured isolates of *B. dermatitidis* from veterinary patients were more likely to be obtained from cases of disseminated disease than humans. This finding suggests that previous associations of genetic group of *B. dermatitidis* with specific clinical phenotypes in humans [14] may also hold true in veterinary infections.

The continued study of *B. dermatitidis* across its range of hosts and geography is key to a better understanding of the life cycle, evolution, and virulence, of this clinically important pathogen.

## Competing interest

All authors declare that they have no competing interests.

## Authors’ contributions

JLA carried out microsatellite typing, retrieved clinical information from veterinary records, and drafted the manuscript. BLS performed genetic and statistical analysis and drafted the manuscript. JKM conceived of and designed the study and drafted the manuscript. All authors read and approved the final manuscript.
